# ROLE OF GAIT SPEED, OLFACTION, AND PSYCHOLOGICAL FACTORS IN COGNITIVE FUNCTION AMONG OLDER ADULTS

**DOI:** 10.1093/geroni/igae098.3050

**Published:** 2024-12-31

**Authors:** Leah Hanson, Kyle Palmberg, Clarissa Howe, Sally Gustafson, Aleta Svitak, Terry Barclay

**Affiliations:** HealthPartners Institute, St. Paul, Minnesota, United States; HealthPartners Institute, St. Paul, Minnesota, United States; HealthPartners Institute, St. Paul, Minnesota, United States; HealthPartners Institute, St. Paul, Minnesota, United States; HealthPartners Institute, St. Paul, Minnesota, United States; HealthPartners, St. Paul, Minnesota, United States

## Abstract

Slowing of gait speed, loss of smell and increased depression, anxiety and stress have been associated with lower cognitive performance among older adults. The Minnesota Memory Project is a longitudinal study of community-dwelling adults 55 and older that investigates lifestyle factors associated with cognitive aging. Annual assessments include the Montreal Cognitive Assessment (MoCA), Alberta Smell Test (AST), gait speed test (GST) and self-report inventories, including the Cognitive Change Checklist (3CL) and measures of social support, resilience, stress, anxiety, and depression. Significant associations were identified between AST and gait scores versus MoCA. Both scores differed significantly between cognitively impaired (MoCA < 26) versus cognitively intact (MoCA ≥26) participants. On average, impaired participant AST scores were significantly lower than intact participants (6.5 ± 3.9 SD, 8.7 ± 3.6 SD) and gait scores were significantly slower (4.0 ± 1.0 SD, 3.65 ± 0.8). A greater number of self-reported cognitive complaints was associated with lower MoCA scores at baseline (p< 0.0001). Participants reporting higher resilience and social support reported significantly fewer cognitive complaints (p< 0.0001), while higher stress, anxiety and depression were associated with more cognitive complaints (all p< 0.0001). Significant effects of olfaction, gait and cognitive complaints on future cognitive decline were also identified. A one-unit increase in AST score was associated with an 11% decrease in the hazard of cognitive impairment within 5 years. A one-unit increase in the GST score was associated with a 60% increase. Screening that includes gait speed, olfaction and subjective cognitive complaints could identify those at risk for future cognitive decline.

